# Individual trajectories of asthma, obesity and ADHD during the transition from childhood and adolescence to young adulthood

**DOI:** 10.25646/7913

**Published:** 2021-04-07

**Authors:** Laura Krause, Felicitas Vogelgesang, Roma Thamm, Anja Schienkiewitz, Stefan Damerow, Robert Schlack, Stephan Junker, Elvira Mauz

**Affiliations:** Robert Koch Institute, Berlin Department of Epidemiology and Health Monitoring

**Keywords:** ASTHMA, OBESITY, ADHD, TRANSITION, YOUNG ADULTHOOD, KIGGS

## Abstract

The German Health Interview and Examination Survey for Children and Adolescents (KiGGS) provides comprehensive and reliable data on the health situation of the upcoming generation. The KiGGS cohort accompanies participants from the KiGGS baseline study (2003–2006) into adulthood. Until now, two follow-up surveys of the cohort have been implemented with KiGGS Wave 1 (2009–2012) and KiGGS Wave 2 (2014–2017). In KiGGS Wave 2, the cohort was supplemented by the in-depth study ‘Family and care-specific factors influencing the development, trajectories and effects of mental disorders (especially ADHD), obesity and allergic diseases (especially asthma)’. One aim of the study was to identify individual trajectories of these health disorders. For this purpose, probabilities for typical transitions from the KiGGS baseline study to KiGGS Wave 2 were calculated. An important result is that many participants who had asthma, obesity or ADHD at KiGGS baseline still had the disease more than ten years later: Over a third still had asthma (35%) or ADHD (37%), and almost half were still affected by obesity (47%). The results point to the need for early preventive measures to stop these potentially chronic diseases from developing in childhood and adolescence.

## 1. Introduction

The majority of children and adolescents in Germany grow up healthy [1]. However, some of them are affected by chronic diseases. In most cases, these not only entail the need for regular health care, but are also accompanied by general and disease-specific stresses that are associated with special psychosocial demands for those affected and their families [2, 3]. The prevalence of chronic conditions in childhood and adolescence has increased worldwide [4]. In Germany, around every sixth girl and boy between the age of 0 and 17 years has a chronic disease [5]. A proportion of these individuals continues to be affected by the condition into adulthood [3].

Bronchial asthma is the most common chronic disease in childhood and adolescence [6]. Asthma is an inflammatory illness of the airways in which the bronchi overreact to physical, chemical, pharmacological or immunological stimuli (hyperreactivity) leading to variable narrowing of the airways (obstruction) [7]. Typical symptoms include whistling noises when breathing (wheezing), coughing, chest tightness and shortness of breath, all of which can be expressed in varying intensities and frequencies. In the majority of cases, asthma among children and adolescents is caused by allergies [8]. Allergic asthmatic reactions result from allergic sensitisations of the immune system. These conditions are often very stressful for children and adolescents and their families and can have an impact on their emotional and physical well-being, as well as on schoolwork and personal relationships [9].

**The KiGGS study**

The German Health Interview and Examination Survey for Children and Adolescents

**Data owner:** Robert Koch Institute

**Aim:** Providing reliable information on health status, health-related behaviour, living conditions, protective and risk factors, and health care among children, adolescents and young adults living in Germany, with the possibility of trend and longitudinal analyses

**Study design:** Combined cross-sectional and cohort study

**KiGGS survey waves:**

- KiGGS baseline study (2003–2006) interview and examination survey
- KiGGS Wave 1 (2009–2012) interview survey
- KiGGS Wave 2 (2014–2017) interview and examination survey

**KiGGS cross-sectional study**

**Population:** Children and adolescents with permanent residence in Germany

**Age range:** 0–17 years

**KiGGS cohort study**

**Sampling:** Re-invitation of everyone who took part in the KiGGS baseline study (n = 17,641) and who was willing to participate in a follow-up

**Age range KiGGS Wave 1:**

6–24 years (n = 11,992)

**Age range KiGGS Wave 2:**

10–31 years (n = 10,853)

More information is available at www.kiggs-studie.de/english

The frequent occurrence of obesity in childhood and adolescence is a global health problem and a major public health challenge for the 21st century [10]. Compared with their peers of normal weight, children and adolescents with obesity have reduced health-related quality of life and are at greater risk of increased blood pressure as well as lipid and glucose metabolism disorders [11, 12]. Furthermore, obesity among children and adolescents is associated with a greater likelihood of type 2 diabetes, high blood pressure and cardiovascular diseases in adulthood [13]. Children and adolescents with obesity may also face discrimination, bullying and stigmatisation due to their body weight, and this can have long-term consequences for their mental health [14, 15].

Attention deficit/hyperactivity disorder (ADHD) is the most commonly diagnosed behavioural disorder among children and adolescents, especially boys [16, 17]. The core symptoms of ADHD are inattention, hyperactivity and impulsivity. These symptoms usually begin in childhood, last longer than six months, occur in several areas of life, lead to noticeable suffering and severely limit a person’s everyday life [18]. The disorder can affect cognitive, academic and social development and pose major challenges to the family and social environment [16]. Children and adolescents with ADHD are also at higher risk for comorbid mental disorders and other health risks, such as substance use [19].

Data on chronic diseases in childhood and adolescence are regularly collected in the German Health Interview and Examination Survey for Children and Adolescents (KiGGS). The KiGGS baseline study (2003–2006) was the first to provide population-based, nationwide representative results on the prevalence of asthma, obesity and ADHD among girls and boys in Germany. KiGGS Wave 2 (2014–2017), conducted around ten years later, provides the most current data available. The data show that 3.5% of 0- to 17-year-olds had asthma or were taking asthma medication in the twelve months prior to the survey, [20] and that 5.9% of 3- to 17-year-olds are obese [21]. Compared to the KiGGS baseline study, the prevalences of asthma (3.2%) and obesity (6.3%) have not changed significantly and remain on a high level – especially obesity. In contrast, parent-reported ADHD diagnoses declined over the decade between the KiGGS baseline study (5.3%) and KiGGS Wave 2 (4.4%) [22].

Data collected from repeated interviews and examinations of participants who had previously taken part in the KiGGS baseline study enable analyses of health developments, trajectories and transitions to be conducted at the individual level (the longitudinal component of KiGGS) [23]. This data can be used to answer the question of the extent to which chronic diseases develop, decline or persist over a period of a good ten years (Info box). Using KiGGS cohort data, this article describes individual trajectories of asthma, obesity and ADHD during the transition from childhood and adolescence to young adulthood.

**Info box**

**Incidence, remission and persistence**

Longitudinal data can be used to illustrate individual trajectories and developments over time. In the course of time, a new occurrence (incidence), an improvement (remission) or no change (persistence) of a condition can be observed.

**Incidence:** At the beginning of the observation, there are individuals who do not have a chronic disease, but they develop one during the observation period.

**Remission:** At the beginning of the observation, there are individuals who are affected by a chronic condition, but no longer did so during the observation period.

**Persistence:** At all observation periods, individuals have a chronic disease.

## 2. Methodology

### 2.1 Data base: the KiGGS cohort

**Figure 1:**
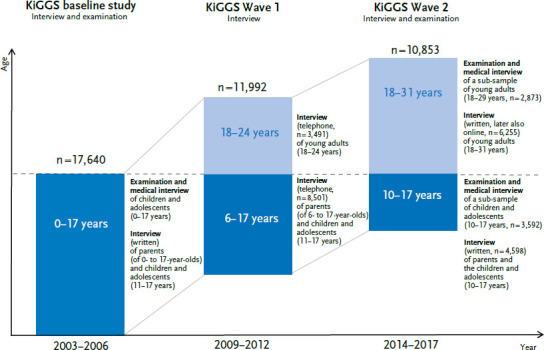
Study design of the KiGGS cohort Source: Own diagram, adapted from Mauz et al. [23]

The KiGGS study is a key source of information for comprehensive and reliable data on the health situation of the upcoming generation [24]. In the longitudinal component – the KiGGS cohort [23] – 17,640 children and adolescents aged between o and 17 years who took part in the KiGGS baseline study (2003–2006; interview and examination survey) were interviewed and examined into young adulthood. The first follow-up took place in KiGGS Wave 1 (2009–2012) as a telephone survey [25], while the second follow-up – KiGGS Wave 2 (2014–2017) – was again conducted as an interview and examination survey [26] (Figure 1). KiGGS Wave 2 was supplemented by the in-depth study ‘Family and care-specific factors influencing the development, trajectories and effects of mental disorders (especially ADHD), obesity and allergic diseases (especially asthma)’. One aim of the in-depth study was to identify trajectory patterns of these health disorders during the transition from childhood and adolescence to young adulthood. In the following, results based on the two interview and examination surveys – KiGGS baseline study and KiGGS Wave 2 – are presented.

### 2.2 Study sample

The initial sample of the KiGGS cohort includes the 17,640 children and adolescents aged between o to 17 years who took part in the KiGGS baseline study [27]. All participants from the KiGGS baseline study who agreed to be re-contacted were invited to KiGGS Wave 2, regardless of their participation in KiGGS Wave 1. Individuals who had moved abroad or who could not be found already at the time of KiGGS Wave 1 were excluded from the invitation. At the time of KiGGS Wave 2, the cohort participants were aged between 10 and 28 years. Due to adjustments in the recruitment process, a small number of adults were already between 29 and 31 years. A total of 10,853 persons (61.5% of the baseline sample) participated in the interviews for the cohort in KiGGS Wave 2. For 6,465 participants of the baseline sample (36.6%), additional examination data are available in KiGGS Wave 2 [27].

### 2.3 Indicators for trajectory description: asthma, obesity and ADHD

The KiGGS baseline study and KiGGS Wave 2 collected data from parents about whether their children had been physician-diagnosed with asthma (ever in their life). Furthermore, it was asked whether the condition occurred in the twelve months before the survey, and whether asthma medication was used in the twelve months before the survey. This information can be used to estimate lifetime and 12-month prevalence of asthma for both surveys [20]. This article focuses on the 12-month prevalence of asthma, comprising both medical diagnoses as well as symptoms and/or medication use in the preceding twelve months prior to the survey.

The analyses for obesity are based on standardised measurements of body height and weight taken from the examinations conducted for the KiGGS baseline study and KiGGS Wave 2 [21]. Body mass index (BMI) is calculated using the ratio of body weight to height squared (kg/m^2^) [28]. Since the relationship between body height and weight changes due to growth in childhood and adolescence, no uniform cut-off can be used to define obesity for children and adolescents across all age groups. Instead, BMI percentile curves are used to classify an individual value which is considered relative to the BMI distribution in a defined group (reference population), considering age and gender. In Germany, obesity is defined using the Kromeyer-Hauschild percentile curves [29, 30]. Children and adolescents are classified as obese if their BMI is above the 97th percentile of the reference population after having accounted for the person’s age and sex. Percentiles for adults were also published in Germany in 2015 [29]. Therefore, this percentile-based definition according to Kromeyer-Hauschild [29] was also used for the adult cohort in order to avoid methodological changes in the transition from adolescence to young adulthood. Children under two years of age were excluded from the analyses, because obesity is not defined as a disease at this age [31].

The indicator for ADHD is the parent-reported ADHD diagnosis which was ever made by a physician or psychologist [16, 32]. Participants aged 18 or above reported this diagnosis themselves. Although this information does not indicate whether the diagnoses reported in KiGGS were made in accordance with guidelines, the indicator proved reliable for children and adolescents aged between 3 and 17 years in an international study [33]. If an ADHD diagnosis was reported both in the KiGGS baseline study and in KiGGS Wave 2, this article refers to the stability of the diagnosis report. The present indicator cannot be used to make any statements about the persistence of a clinical ADHD. Furthermore, no conclusions can be made about remission of ADHD, since the indicator used here depicts lifetime prevalence. In addition, it is questionable whether a neurobiological disorder such as ADHD can lead to remission or whether only symptom relief occurs.

### 2.4 Statistical analysis

The statistical analyses of transition probabilities are based on data from 8,594 persons from the age of three years for asthma; 5,447 persons from the age of two years for obesity and 6,773 persons from the age of three years for ADHD. Transition probabilities between the KiGGS baseline study (2003–2006) and KiGGS Wave 2 (2014–2017) were calculated for the incidence, remission and persistence/stability of asthma, obesity and ADHD. Since the two surveys took place about ten years apart, the cumulative ten-year incidence, ten-year remission and ten-year persistence/stability are calculated. In the following, the terms incidence, remission and persistence or stability are used for better readability.

All percentages are reported with 95% confidence intervals (95% CI). They represent the statistical uncertainty of the estimator. For this reason, percentages with two digits are presented without additional decimal place (e.g. 35%). A figure of 34.7% implies a precision, which cannot be concluded from the data. Single-digits frequencies, however, are rounded to one decimal place so as to provide two significant digits (e.g. 2.4%). This approach is supported by the fact that statistical uncertainty is lower at the borders of the distribution. A statistically significant difference between groups is assumed when p-values are smaller than 0.05.

Weighting factors are used to compensate for possible bias in the sample due to selective re-participation [26, 34]. The analyses are carried out using SAS (version 9.4) and Stata (version 15.1) with survey procedures that account for the study design and weighting.

With regard to asthma and ADHD, all analyses are carried out separately for girls/women and boys/men in order to provide sex-specific results. However, age-dependent development is shown for obesity as there is a relation between body growth and puberty. These results build on the findings regarding the individual trajectories of obesity among preschool children published at the KiGGS Symposium in 2018 [35].

## 3. Results

Of the children and adolescents aged three years or above without asthma at KiGGS baseline, 3.4% (95% CI: 2.9–4.0) had reported a medical diagnosis of asthma and current symptoms and/or asthma medication around ten years later (incidence). Of those who were asthmatic at KiGGS baseline, 65% (95% CI: 56–73) were free of symptoms and/or medication around ten years later (remission). This means that slightly more than one third (35%, 95% CI: 27–44) were still affected by asthma and/or were using asthma medication when KiGGS Wave 2 was conducted (persistence).

**Figure 2:**
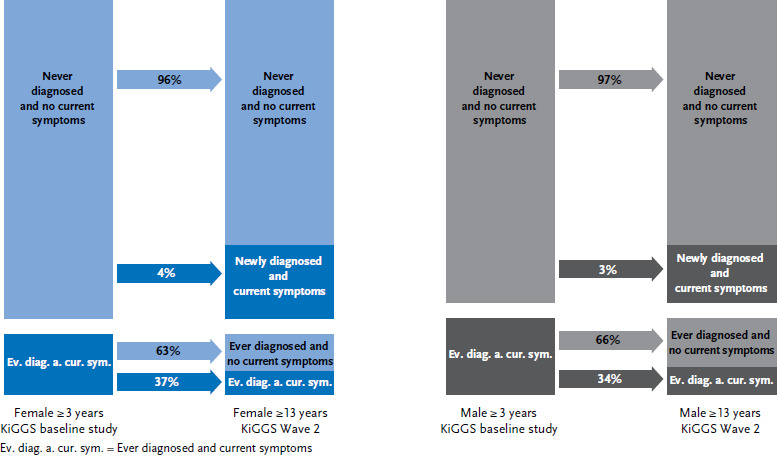
Individual 10-year trajectory of asthma by sex (n = 4,636 female, n = 3,958 male) Source: KiGGS baseline study (2003-2006), KiGGS Wave 2 (2014-2017)

Figure 2 shows the probabilities of asthma disease during the transition from childhood and adolescence to young adulthood by sex. No statistically significant difference was identified between the sexes with regard to the incidence, remission and persistence of asthma.

With regard to obesity, Table 1 shows that around 5% of children and adolescents aged two years and older who were not affected by obesity in the KiGGS baseline study developed obesity over the following ten years (incidence). Just over half of the children and adolescents who were affected by obesity in the KiGGS baseline study were no longer obese ten years later (remission). Consequently, a little less than half of the participants remained in this category when KiGGS Wave 2 was conducted (persistence).

The results stratified by age show that 6.7% of children aged between two and six years in the KiGGS baseline study developed obesity in the following ten-year period (incidence). This applies to only 3.8% in the oldest age group of 14- to 17-year-olds (Table 1). Thus, the incidence of obesity tends to decrease with increasing age. However, it should be noted that the proportion of children with obesity in the KiGGS baseline study also increases with age. Adolescents who are not affected by obesity by the age of 14 and 17 years are less likely to develop obesity than children who are not affected by obesity between the ages of two and six.

A little more than one third of 2- to 6-year-old children with obesity in the KiGGS baseline study were no longer obese ten years later; among 14- to 17-year-olds is the proportion with 50% higher (remission). This means that around two thirds of 2- to 6-year-old children with obesity in the KiGGS baseline study were still affected by obesity around ten years later (persistence).

Table 2 shows that for 2.4% of participants aged three years or above whose parents did not report an ADHD diagnosis at the time of the KiGGS baseline study, an ADHD diagnosis was reported for the first time during the following ten years (incidence). This was more often true for male than for female participants. For slightly more than one third of the children and adolescents with parent-reported ADHD diagnosis at KiGGS baseline, a diagnosis was reported again in KiGGS Wave 2. With regard to the stability of the ADHD diagnosis report, there are no differences by sex.

## 4. Discussion

Data from the KiGGS study show that a notable proportion of children and adolescents living in Germany are affected by asthma, obesity or ADHD [20–22]. The aim of the present article was to quantify patterns of these selected chronic diseases in the transition from childhood and adolescence to young adulthood.

Data from the KiGGS cohort indicate that slightly more than one third of the children and adolescents who had a medical asthma diagnosis at KiGGS baseline and who reported symptoms and/or had taken medication for the condition in the preceding twelve months prior to the survey were still affected by asthma when KiGGS Wave 2 was conducted. 3.4% of children and adolescents without asthma at KiGGS baseline had been medically diagnosed with asthma for the first time during the following ten years and reported current symptoms and/or current medication. It is difficult to compare these results with those of other national or international longitudinal studies in children and adolescents. There are major differences between the studies in study design, specific study question, inclusion criteria (e.g. with regard to age), follow-up time and baseline time. Especially in Europe, several population-based birth cohort studies have been established so far, aiming, among other things, to investigate asthma trajectories [36–38]. In Germany, the MAS study (Multicentre Allergy Study; started in 1990 [39]), the GINI (plus) study (German Infant Nutritional Intervention plus environmental and genetic influences on allergy development) and the LISA (plus) study (Influence of Life-style Factors on Development of the Immune System and Allergies in East and West Germany; both started in 1995 [40]) should be mentioned. In contrast to the KiGGS cohort, which is based on a representative sample for Germany, it was particularly important for the intervention arms of the birth cohort studies to include newborns with an increased allergy risk. In the MAS study, asthma trajectories have been estimated stratified by family history of the condition. Children whose parents have allergies – asthma, allergic rhinitis or atopic dermatidis – were found to have a cumulative 20-year incidence of about 40%; if only one parent had an allergy, this rate was 25%. Among children of nonallergic parents, the cumulative 20-year incidence reached a plateau of just over 10% in early adolescence [41]. The GINI (plus) and LISA (plus) studies, which used data provided by parents on medical diagnoses, found that the prevalence of asthma increased during the first ten years of life. A cumulative 10-year asthma incidence of approximately 3% to 4% can be derived from the two studies [40]. In line with the KiGGS study, the results on individual asthma trajectories show that a considerable proportion of children and adolescents become asthmatics and remain affected by the condition over a long period of their lives and continue to require therapy. Therefore, and also in view of the fact that asthma is often associated with other allergic diseases such as allergic rhinitis and atopic dermatitis, the recently published National Asthma Care Guideline specifically addresses children and adolescents [42].

Only a few prospective studies have examined the individual trajectories of obesity in children and adolescents in Germany [43–45]. These studies indicate a high level of persistence and incidence and a low level of remission among primary school children. Thus, obesity among this age group often persists through adolescence into adulthood. In addition, many children and adolescents develop obesity with increasing age, while only a relatively small number of children with obesity in primary school return to a normal weight. A study from the United States indicates that children who are affected by obesity when they start school do not tend to be affected temporarily [46]. The transition between normal weight, overweight and obesity is more flexible among preschool children, but even at this age, only a small number of children with obesity returns to normal weight later in life. The KiGGS cohort confirms these findings and indicates that obesity among children of preschool age often persists into adolescence and young adulthood. Consequently, preventive measures should also be targeted at children of nursery and primary school age [21]. A high level of persistence has been found for obesity among school-aged children, although incidence decreases with age [47, 48]. As such, obesity is more likely to persist with increasing age, but the number of adolescents with incident obesity does not increase in the same extent as in younger ages. This is also confirmed by the KiGGS cohort: overall, the proportion of children and adolescents with obesity increases but the proportion of incident cases seems to decrease with increasing age. Furthermore, the results of the KiGGS cohort suggest that obesity persistence tends to decrease and remission tends to increase up to puberty. Thus, the time frame until puberty seems to be reasonable to prevent obesity (for obesity prevention see also the current S3 guideline ‘Therapy and prevention of obesity in childhood and adolescence’ [49]).

The trajectory of ADHD, especially during the transition to young adulthood, has so far been little studied in Germany. Data from the KiGGS cohort show that 2.4% of the children and adolescents who participated in the KiGGS baseline study were diagnosed with ADHD for the first time during the next ten years. As expected, for more than one third of the children and adolescents with an ADHD diagnosis report at the time of the KiGGS baseline study, a corresponding diagnosis was also reported a good ten years later. It can be assumed that the group of participants with a stable diagnostic report is also more affected by persistent ADHD symptoms and that the probability of persistent trajectories is increased in this group. This is supported by the fact that this group had higher symptom burdens in psychopathological screening in both the KiGGS baseline study and KiGGS Wave 1 [50]. An American study that examined the stability of ADHD diagnoses over a comparable period of eleven years came to similar stability rates of clinical ADHD diagnoses as the KiGGS cohort with the ADHD diagnosis report, reporting a persistence rate of 35% [51].

The KiGGS study, as a nationwide representative health survey, provides valuable, unique data for the assessment of disease incidence for common chronic diseases among children and adolescents such as asthma, obesity and ADHD at the population level. An important finding of the cohort is that many participants in the KiGGS baseline study (2003–2006) with asthma, obesity or ADHD were still affected by the condition around ten years later. Slightly more than one third of those affected by asthma (35%) or ADHD (37%) reported the same condition in KiGGS Wave 2 (2014–2017); for obesity, it was even almost half (47%). On the other hand, only a few participants of the KiGGS baseline study developed one of these chronic diseases during the following ten years (2.4% ADH D, 3.4% asthma, 5.1% obesity). These results point to the need for early preventive measures to ensure that children and adolescents do not develop these potentially chronic diseases.

In order to obtain an even more detailed picture of disease incidence, it may be useful in the future to link the data from the KiGGS study to other (secondary) data, for example data from statutory health insurance. For the KiGGS cohort, once case definitions in the routine data have been validated [52], the data could be used to associate complex trajectories across the lifespan with baseline conditions in childhood and adolescence and health outcomes in adulthood.

## Key statements

- Just over a third of children and adolescents with asthma at KiGGS baseline were still affected by the condition and/or were still using asthma medication a good ten years later.
- Almost 4% of children and adolescents without asthma at KiGGS baseline had been medically diagnosed with asthma for the first time during the following ten years and reported current symptoms and/or current asthma medication.
- About half of the children and adolescents with obesity at KiGGS baseline were no longer affected by obesity around ten years later.
- Obesity among preschool aged children often persists into adolescence and young adulthood.
- 2.4% of children and adolescents who had not been diagnosed with ADHD at KiGGS baseline were reported with a diagnosis for the first time a good ten years later.

## Figures and Tables

**Table 1:** 10-year individual trajectory of obesity (n = 5,447) Source: KiGGS baseline study (2003-2006), KiGGS Wave 2 (2014-2017)

Age at KiGGS baseline	Cumulative 10-year incidence		Cumulative 10-year remission		Cumulative 10-year persistence	
	%	(95% CI)	%	(95% CI)	%	(95% CI)
**Total**	**5.1**	**(4.3–6.0)**	**53**	**(44–61)**	**47**	**(39–56)**
2–6 years	6.7	(5.3–8.4)	35	(21–52)	65	(48–79)
7–10 years	4.9	(3.4–7.0)	57	(44–70)	43	(30–57)
11–13 years	4.5	(2.8–7.2)	63	(44–78)	37	(22–56)
14–17 years	3.8	(2.4–6.0)	50	(33–66)	50	(34–67)

CI = confidence interval

**Table 2:** Incidence and stability of ADHD lifetime diagnoses by sex (n = 3,742 female, n = 3,031 male) Source: KiGGS baseline study (2003–2006), KiGGS Wave 2 (2014–2017)

	Cumulative 10-year incidence		Cumulative 10-year stability	
	%	(95% CI)	%	(95% CI)
**Total**	**2.4**	**(1.9–3.0)**	**37**	**(28–47)**
Female	1.3	(0.9–2.0)	36	(22–53)
Male	3.6	(2.7–4.7)	38	(27–50)

CI = confidence interval
